# DIFFERENCES IN HEALTH TRAJECTORIES BETWEEN CANCER SURVIVORS AND NONCANCER OLDER ADULTS

**DOI:** 10.1093/geroni/igac059.587

**Published:** 2022-12-20

**Authors:** Minzhi Ye, Eva Kahana, Gary Deimling, Adam Perzynski, Kurt Stange

**Affiliations:** Kent State University, Cleveland, Ohio, United States; Case Western Reserve University, Cleveland, Ohio, United States; Case Western Reserve University, Cleveland, Ohio, United States; MetroHealth and Case Western Reserve University, Cleveland, Ohio, United States; Case Western Reserve University Center for Community Health Integration, Cleveland, Ohio, United States

## Abstract

The current study addresses how cancer and aging influence older adults' health trajectories differently. The unique cross-sequential design allowed the study to compare the health changes between long-term (5 years +) older cancer survivors (breast, prostate, and colorectal cancer) and demographic-matched older adults without a history of cancer in the same geographic area within the same period by merging two longitudinal studies. The study also captured comprehensive information on health disparities over time. General linear models were employed. The findings showed that neither previous cancer experience nor aging affects health trajectories in later life. Conversely, comorbidities, being African American, female, having less than a college degree, and living alone significantly decreased the health trajectory in later life for all older adults. Moreover, when compared to other groups, older African American cancer survivors reported low scores in self-reported health after controlling for other conditions.

